# Who is That? Brain Networks and Mechanisms for Identifying Individuals

**DOI:** 10.1016/j.tics.2015.09.002

**Published:** 2015-12

**Authors:** Catherine Perrodin, Christoph Kayser, Taylor J. Abel, Nikos K. Logothetis, Christopher I. Petkov

**Affiliations:** 1 Department of Physiology of Cognitive Processes, Max Planck Institute for Biological Cybernetics, 72076 Tübingen, Germany; 2 Institute of Behavioural Neuroscience, University College London, London, WC1H 0AP, UK; 3 Institute of Neuroscience and Psychology, University of Glasgow, Glasgow, G12 8QB, UK; 4 Department of Neurosurgery, University of Iowa, Iowa City, IA 52242 USA; 5 Division of Imaging Science and Biomedical Engineering, University of Manchester, Manchester, M13 9PT, UK; 6 Institute of Neuroscience, Newcastle University Medical School, Newcastle upon Tyne, NE2 4HH, UK

**Keywords:** face, voice, multisensory, identity, human, primate, temporal lobe

## Abstract

Social animals can identify conspecifics by many forms of sensory input. However, whether the neuronal computations that support this ability to identify individuals rely on modality-independent convergence or involve ongoing synergistic interactions along the multiple sensory streams remains controversial. Direct neuronal measurements at relevant brain sites could address such questions, but this requires better bridging the work in humans and animal models. Here, we overview recent studies in nonhuman primates on voice and face identity-sensitive pathways and evaluate the correspondences to relevant findings in humans. This synthesis provides insights into converging sensory streams in the primate anterior temporal lobe (ATL) for identity processing. Furthermore, we advance a model and suggest how alternative neuronal mechanisms could be tested.

## Trends

Our ability to identify unique entities, such as specific individuals, appears to depend on sensory convergence in the anterior temporal lobe.

However, the neural mechanisms of sensory convergence in the anterior temporal lobe are unclear.

Alternative accounts remain equivocal but could be tested by better bridging the findings in humans and animal models.

Recent work in monkeys on face- and voice-identity processes is helping to close epistemic gaps between studies in humans and animal models.

We synthesize recent knowledge on the convergence of auditory and visual identity-related processes in the anterior temporal lobe.

This synthesis culminates in a model and insights into converging sensory streams in the primate brain, and is used to suggest how the neuronal mechanisms for identifying individuals could be tested.

## Missing Pieces in Identity Processes

Certain individuals are unmistakable by their visual face or auditory voice characteristics, others by their smell or how they move. Identifying an individual, or any other unique entity, is an instance of the general problem of object identification, which is a process occurring at different levels of categorization (e.g., basic or subordinate). At a basic level, identifying objects relies on recognizing the categorical features of the object class; social animals can also perceptually categorize species membership, social rank, body size, or age 1, 2. However, individuals are unique entities identified by more subtle within-category differences, referred to as ‘subordinate level’ identification. For example, while a human or monkey might be content to eat ‘a’ banana, social situations critically depend on identifying specific individuals to avoid or interact with. Identifying unique concrete entities, such as specific individuals, can be achieved by input from several sensory modalities, whereas the sound of a banana falling onto the ground may be indistinguishable from the sound of another fruit falling.

The nature of the multisensory computations underlying individual identification in the brain remains unclear. Here, we consider two scenarios: it could be that each sensory input is sufficient to activate an identity-specific neuronal representation. In this case, unique individual identification likely relies on **amodal** (see Glossary) or modality-independent convergence sites, whose neural representations can be driven by any sensory input. We refer to this as an ‘or gate’. Alternatively, identification may emerge from the synergistic interplay of all available incoming signals that collectively shape the neural representation. In this case, missing input from one sensory stream will alter the neural representations at the site of convergence. We refer to this as a ‘synergistic’ process, which could be additive or nonadditive 3, 122. Pursuing the underlying mechanisms, whatever they may be, and their impact on behavior will reveal how neural activity is used to identify individuals and unique entities.

Initial insights into the regions underlying the identification of individuals were provided by lesion and **neuroimaging** studies 4, 5, 6, 7. Such work revealed a distributed network of brain regions engaged in extracting different types of sensory feature, such as faces [8]. Lesion studies show that damage to face-selective regions in occipital, fusiform cortex and the **ATL** can impair face perception, a disorder known as ‘**prosopagnosia**’ 9, 10, 11, 12. The analogous auditory disorder affecting voices (‘**phonagnosia**’) [13] can arise from damage to parts of the same temporal lobe network involved in prosopagnosia, although the heterogeneity in lesion size and location across patients makes more detailed distinctions difficult 4, 12, 14. Lesions of the language-dominant (left) ATL are associated with a decline in the ability to name both famous faces and famous voices 15, 16. Naming a person involves lexical retrieval, which depends on language-related processes in frontal, temporal, and parietal regions around the Sylvian sulcus [17], including the ATL 18, 19, 20.

However, current accounts of the neural processes involved in assessing identity remain equivocal. The most common approaches can identify the large-scale neural substrates but provide limited insights into the overlap, segregation, and form of neuronal representations involved in identity processes, because neuroimaging approaches measure either surrogates of neuronal activity or large-scale neural responses. Consequently, there is a need for direct measures of localized neuronal computations to resolve alternative accounts. Direct neuronal recordings (depth electrode recordings or electrocorticography) in human patients being monitored for neurosurgery can inform on neuronal function in localized regions in the human brain, while work in animal models can describe neuronal processes at multiple scales directly from the regions of interest and offers greater specificity in neuronal manipulation (activation and/or inactivation). However, until recently the animal work had not kept apace. The current literature in humans considers multisensory interactions and convergence as a research priority, with studies often collecting data from at least two sensory modalities 4, 14. The human work also highlights the advantage of combining visual (face) and auditory (voice) input for identity recognition 21, 22. By contrast, neuronal-level studies in animal models are usually restricted to studying one sensory modality (e.g., face-identity processes in the visual system). In that respect, recent findings from auditory voice identity-related neuronal studies in monkeys may help the integration of human and nonhuman animal work and increase our understanding of the organization of identity processing in the brain.

Here, we briefly overview two alternative neurocognitive models of identity processing developed in humans. We then review recent studies on voice- and face-identity processes and multisensory pathways conducted in nonhuman primates and evaluate the correspondences to relevant findings in humans. From this synthesis, we propose a model of primate ATL function for identity-related processes and use it to identify imminent gaps in our understanding. We conclude by suggesting how alternative neuronal mechanisms could be tested.

## Human Models of Identity Perception: What Are the Neuronal Mechanisms?

Current theoretical models developed from human studies of face- and voice-identity perception have suggested that the related auditory and visual streams converge in the ATL 4, 5, 6, 7, 12, 23. Convergence in the ATL has also been independently articulated in lesion studies of semantic disorders, where neurosurgical resection, stroke, or degeneration of the ATL (bilaterally or unilaterally [24]) affects a person's ability to name or recognize an individual by seeing their face or hearing their voice 18, 19, 25, 26, 27.

Two prominent, not mutually exclusive, models are as follows. A ‘distributed-only’ model proposes that the sensory features important for recognizing an individual engage distinct brain regions, interconnected into a network 4, 18, 19. This model does not require amodal convergence sites because the interconnectivity allows inputs from different sensory modalities to influence the collective network-wide processes. Damage to any node in a distributed network will selectively disrupt the key contribution of that node and influence, but not necessarily preclude, the function of the rest of the network. For instance, a lesion of voice-**sensitive** regions might result in phonagnosia and affect voice–face multisensory interactions, but will not disrupt the ability to identify an individual with inputs from the preserved sensory modalities. Another ‘distributed-plus-hub’ model (or related ‘hub-plus-spoke’ models) for identity processing not only contains distributed processes, but also features the ATL as a key hub or convergence site whose function is amodal 4, 5, 6, 7, 18, 19, 23. Crucially, the function of a damaged amodal process cannot be recovered by the rest of the network (for a computational model, see [25]).

Both models rely on **multisensory convergence** sites, but differ in the processing at these sites. In this paper, we take this a step further to suggest neuronal mechanisms that could be tested even at the level of single neurons. For instance, multisensory convergence in the ATL as an amodal process suggests an ‘or gating’ function where one or another synaptic input is sufficient to result in neuronal depolarization. The alternative mechanism is a synergistic interaction of the available multisensory inputs, such that the form of neuronal representations depends on the combination of the different available inputs. It is also possible that the ‘or gating’ occurs as a side product of the converging synergistic multisensory neural process being assigned a top-down label.

Thereby, different scientific lines have converged on questioning the neural multisensory interactions in ATL sites and the identity-related processes that they support. Although animal models cannot directly address questions of interest for lexical retrieval, since naming relies on human language, work in nonhuman animals can clarify which identity processes in the ATL are evolutionarily conserved and the cognitive functions that they support (e.g., perceptual awareness, conceptual knowledge; see Outstanding Questions). Recent developments now allow better bridging gaps between the work in humans and other animals.

## Face and Voice Regions in Humans and Other Animals

First, face-sensitive neurons were identified in the monkey inferior temporal (IT) cortex as neurons that respond more strongly to face than to nonface objects 28, 29. Subsequently, neuroimaging studies revealed face-category preferring regions in the human fusiform gyrus (FG) and occipital areas 8, 30, 31 and in the monkey fundus and inferior bank of the superior temporal sulcus (STS) 32, 33, 34, 35, 36. In the auditory modality, voice-sensitive regions have only recently been identified in humans and other animals.

Auditory studies in animal models have shown that neuronal responses typically become increasingly selective for complex sound features along the auditory processing hierarchy 37, 38, 39, 40, 41, 42, and that the ventral-stream pathway processing ‘what’ was vocalized in primates involves auditory cortex 43, 44, the anterior superior temporal gyrus (aSTG) 38, 45, temporal polar cortex [46], anterior insula [47], and ventrolateral prefrontal cortex (vlPFC) 48, 49. To more directly study ‘who’ rather than ‘what’ was vocalized requires using stimuli that differ in **voice content**.

**Figure 1:**
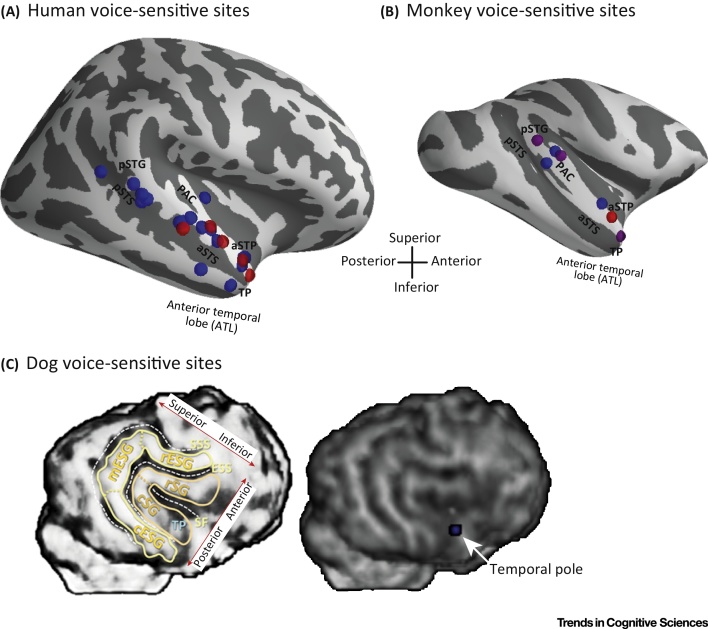
Temporal Lobe Voice Areas in Humans, Monkeys, and Dogs. (A) Voice category-sensitive sites (voice versus nonvoice sounds; blue) in the human temporal lobe or those that are voice-identity sensitive (within category; red). The identified sites are projected onto the surface using pySurfer software^i^ and correspond to the identified peak of activity clusters reported in 50, 57, 58, 59, 60, 76, 117. This representation focuses only on the temporal lobe and the right hemisphere, although, as the original reports show, the left hemisphere also has temporal voice-sensitive regions. For a recent probabilistic map of human voice-category sensitive regions, see [87]. (B) Summary of voice-category and voice-identity sensitive sites in the macaque temporal lobe, obtained from peak activity clusters reported in [52]. Also shown are vocalization-sensitive peak responsive sites (purple) reported in other macaque neuroimaging studies 46, 118, 119. (C) Voice-category sensitive areas in the brains of domesticated dogs [53], showing a cluster in the anterior temporal lobe. Abbreviations: a, anterior; c, caudal (posterior); ESS, ectosylvian sulcus; m, middle; p, posterior; PAC, primary auditory cortex; r, rostral (anterior); rESG, rostral ectosylvian gyrus; SF, Sylvian fissure; SG, Sylvian gyrus; SSS, suprasylvian sulcus; STG, superior temporal gyrus; STP, supratemporal plane; STS, superior temporal sulcus; TP, temporal pole. Images provided by A. Andics (C).

Regions responding more strongly to voice versus nonvoice categories of sounds were first identified in humans with functional magnetic resonance imaging (fMRI) [50] and include regions in the STG and STS (Figure 1A). However, it is known that human voice regions can also strongly respond to or decode speech content [51], raising the concern that voice and speech representations might be functionally interdependent in the human brain and not evident in the same way in the brains of other animals. With the advent of auditory fMRI in nonhuman animals, scientists were able to address this: the comparison of voice versus nonvoice-driven responses showed evidence for evolutionary counterparts to human voice regions in the monkey supratemporal plane (STP) (Figure 1B) [52] and in the temporal lobe of domesticated dogs (Figure 1C) [53].

There are multiple voice category-preferring clusters in the primate STP [52], just as there exist several face category-preferring clusters in more inferior parts of the temporal lobe 33, 34, 54. Yet, complementary findings have now been obtained in both auditory and visual modalities that point to ATL regions being more sensitive to unique identity compared with more posterior temporal lobe regions (face identity: humans 36, 55, monkeys 32, 56; voice identity: humans 57, 58, 59, 60, monkeys [52], Figure 1A,B; infant voice-sensitive regions 61, 62).

## Voice Cells, Face Cells and Multisensory Interactions

**Figure 2:**
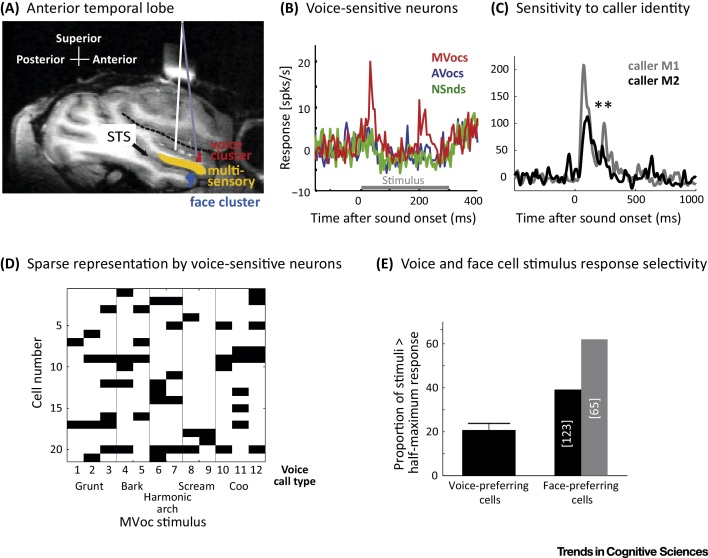
Voice- and Face-Sensitive Neuronal Responses in Monkeys. (A) Targeting approach for recording from the anterior voice identity-sensitive functional magnetic resonance imaging (fMRI) cluster (red). Multisensory cortex in the upper bank of the superior temporal sulcus (STS) is illustrated in yellow. The fundus and the lower bank of the STS can contain face-sensitive clusters (blue, see main text). (B) Voice-sensitive neurons show a categorical response to monkey vocalizations produced by many different callers (MVocs) that is twofold greater than responses to vocalizations from other animals (AVocs) or nonvoice natural sounds (NSounds) [63]. (C) Units sensitive to voice (caller) identity are often found within the pool of voice category-preferring units. Such units show comparable responses to two different vocalizations (here the response to ‘coo’ and ‘grunt’ calls is averaged) but differential responses to individual callers (caller M1 versus M2) [64]. (D) Voice-sensitive neurons respond selectively to a small subset of the stimuli within the conspecific voices. (E) Voice-sensitive cells appear to be more stimulus selective (i.e., respond well to smaller percentages of the presented voices, [63]) compared with face cells, which tend to respond to approximately 55% of the faces within the face stimuli 35, 65, 123. Modified, with permission, from [63] (A,C).

In monkeys, targeted neural recordings in voice identity-sensitive fMRI clusters in the ATL provided the first evidence for voice cells (Figure 2A) [63]. These neurons respond strongly to the category of conspecific voices (Figure 2B) as well as differentially to specific voices within that category (Figure 2C,D) [63]. The neurons in the ATL voice region are also sensitive to auditory features in the vocalizations, such as caller identity (Figure 2C), further qualifying this anterior STP region as a higher-order auditory area [64] and supporting the notion that the ATL is important for identity processes.

However, the functional organization of face- and voice-sensitive clusters in the ATL may not be identical [63]. For example, face cells might be more abundant in fMRI-identified face patches [35] and be less selective to individual static faces (Figure 2E) 35, 65. By contrast, voice cells cluster in modest proportions and respond selectively to a small subset of the presented voice stimuli (Figure 2E); for further discussion see [63]. This high stimulus **selectivity** of auditory ATL neurons is not unexpected [38] and is on a par with the selectivity of neurons in the vlPFC 40, 48. These initial comparisons suggest potential divergences in the neuronal substrates of identity representations in the auditory and visual streams at these processing stages.

**Figure 3:**
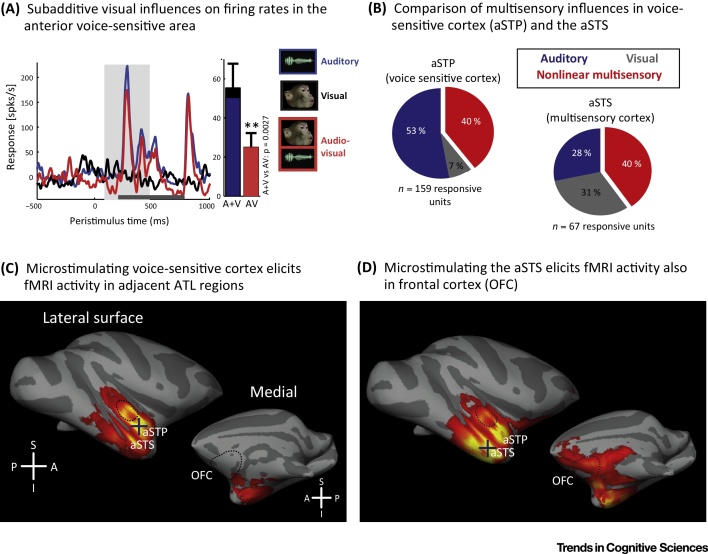
Neuronal Multisensory Influences and Effective Functional Connectivity in the Monkey Brain. (A) Example of a nonlinear (subadditive) multisensory unit in voice-sensitive cortex: firing rates in response to combined audiovisual stimulation (AV, voice and face) significantly differ from the sum of the responses to the unimodal stimuli (A, auditory; V, visual). (B) Neuronal multisensory influences are prominent in voice-sensitive cortex (anterior supratemporal plane; aSTP) but are qualitatively different from those in the anterior superior temporal sulcus (aSTS). For example, aSTS neurons more often display bimodal responses [64]. (C) A study of effective functional connectivity using combined microstimulation and functional magnetic resonance imaging (fMRI) shows that stimulating voice-sensitive cortex (blue cross) tends to elicit fMRI activity in anterior temporal lobe (ATL) regions [81]. (D) By contrast, stimulating the aSTS also elicits fMRI activity in frontal cortex, in particular the orbitofrontal cortex (OFC). Abbreviations: A, anterior; I, inferior; P, posterior; S, superior. Modified, with permission, from [64] (A).

Regarding the nature of multisensory interactions underlying individual identification, there is now substantial evidence for anatomical and functional crosstalk at various stages of the sensory pathways in humans and many other animal species 66, 67, 68, 69, 70, 71, 72, 73. Neuronal responses to voices and dynamic faces have been compared in monkeys between the voice-sensitive anterior (a)STP and the anterior upper-bank of the STS (uSTS) 64, 73, 74, which is part of the multisensory association cortex in primates 64, 66, 68, 72, 73, 75, 76. Anterior uSTS neurons, unlike those in the aSTP, are not particularly sensitive to auditory vocal features [64], which is also the case for more posterior regions of the uSTS 73, 74. By comparison, however, anterior uSTS neurons show a balance of both auditory and visual responses (Figure 3B) and are sensitive to the congruency of the presented voice–face pairings: multisensory influences in the uSTS tend to occur more frequently in response to matching compared with mismatched audiovisual stimuli, such as a monkey face being paired with a human voice. By contrast, aSTP neurons exhibit weak visual-only responses [64]. Also, multisensory influences in the aSTP are less selective for correct face–voice pairings and are qualitatively more similar to those reported in and around primary auditory cortex than they are to those in the STS [70]. These observations are consistent with the evidence for integrative multisensory processes in the human and monkey STS 74, 77, potentially at the cost of decreased specificity of unisensory representations [68]. The results reveal visual modulation in the ATL, but underscore the auditory role of the primate voice-sensitive aSTP, with more robust multisensory integration occurring in the STS.

Several studies have also assessed the timing of neuronal responses relative to oscillatory activity, as a mechanism for routing and prioritizing sensory information [78]. For instance, the latencies of auditory cortical responses decrease when there is a behavioral benefit of a visual face on the reaction time in detecting an auditory voice [79]. Also, neurons in the monkey voice-sensitive aSTP show crossmodal (face-on-voice) phase resetting that can predict the form of multisensory neuronal responses [71]. These phase-resetting effects appear to be more similar to those reported in and around primary auditory cortex than they do to those reported in the STS [72]. Moreover, neurons in the monkey STS show specific patterns of slow oscillatory activity and spike timing that reflect visual category-specific information (faces versus objects) [80]. Taken together, this suggests that the interplay of individual neurons and the local network context shapes sensory representations. Yet, whether oscillatory processes are specifically involved in identity processing or constitute more general computational principles shared across brain regions remains unclear (see Outstanding Questions).

## Interconnectivity between Face and Voice Regions and Other Areas

Recently, the directional effective connectivity of the voice network was investigated using combined microstimulation and fMRI in monkeys, providing insights into voice-related and multisensory processing pathways in the primate ATL [81]. Stimulating a brain region while scanning with fMRI can reveal the synaptic targets of the stimulated site, a presumption supported by the fact that target regions activated by stimulation are often consistent with those identified using neuronal anterograde tractography (e.g., 81, 82).

Surprisingly, microstimulating voice identity-sensitive cortex does not strongly activate PFC, unlike stimulation of downstream multisensory areas in the STS and upstream auditory cortical areas in the lateral belt [81]: the voice-sensitive area in the primate aSTP seems to interact primarily with an ATL network including the uSTS and regions around the temporal pole (Figure 3C). By contrast, stimulating the uSTS results in significantly stronger frontal fMRI activation, particularly in orbital frontal cortex (Figure 3D). These observations suggest that multisensory voice and face processes are integrated in regions such as the uSTS in the ATL before having a strong impact on frontal cortex, providing additional insights to complement those on ATL connectivity 49, 83, 84, 85, 86, 115 and neuronal processes 38, 46, 64.

However, there is a noted distinction between species [52], because human voice-sensitive clusters are often localized in the STS, which in monkeys is classified as multisensory association cortex 3, 66. Interestingly, a recent probabilistic map of human temporal voice areas suggests that anterior voice-sensitive regions are located in the human STG and posterior ones in the STS [87]. Thus, there may be a close correspondence across the species in terms of anterior voice-sensitive clusters and multisensory processes in the STS, although this issue is worth evaluating further (see Outstanding Questions).

Human neuroimaging studies have shown that voice and face regions in the temporal lobe can be respectively influenced by the other modality 88, 89, 90, and are structurally connected to each other [91]. Another study found that the posterior STS bilaterally and the right anterior (a)STS respond preferentially to people-related information regardless of the sensory modality [76], which could be construed as certain human voice regions in the anterior STS being amodal 92, 93. However, it is currently unclear whether and how voice and face regions in the human temporal lobe are interconnected with multisensory regions in the STS and those in the temporal pole or frontal cortex, knowledge that is already available in monkeys. Ongoing efforts could be complemented with more direct measures of local ATL neural responses to voices and faces in humans to compare with **intracranial recordings** in monkeys.

## Human Intracranial Recordings during Face and Voice Naming

**Figure 4:**
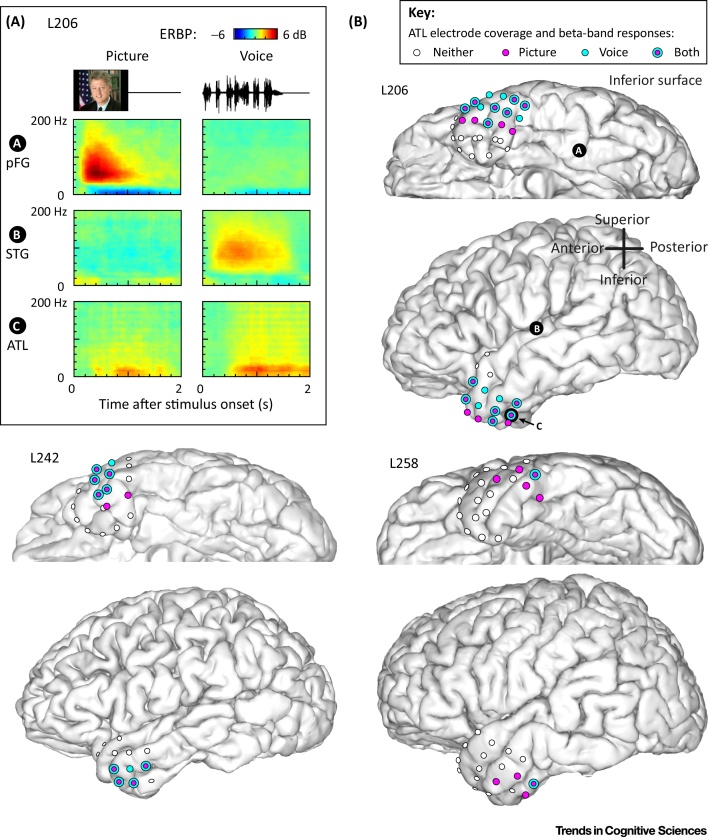
Anterior Temporal Lobe (ATL) Neuronal Recordings in Humans. Intracranial human recordings from several areas in the temporal lobe during an auditory and visual identity naming task. (A) Regions of the ATL are responsive to both a picture and the voice of an individual [26]. By contrast, a visual area in the posterior fusiform gyrus (pFG) responds mainly to the picture, and auditory cortex on the superior temporal gyrus (STG) to the sound. Note that verbal naming followed the period of recording in response to the faces and/or voices as stimuli. (B) Some contacts in the two patients (L206, L242, and L258) show unimodal (picture or voice) responses in the ATL, particularly in the beta band. Other contacts show responses to both. Modified, with permission, from [26].

An earlier study recording from neurons in the medial temporal lobe (MTL) of patients reported highly selective responses to pictures of known celebrities, such as Jennifer Aniston [94]. Recently, several studies have been conducted in human subjects performing voice- and face-naming tasks 26, 92, 95. One group in particular has developed more extensive coverage of the different ATL regions for subdural cortical recordings 26, 96. Using a voice- or face-naming task while recording local field potentials revealed strikingly similar neuronal responses in the ATL regardless of the form of the sensory input, auditory or visual (Figure 4). By contrast, electrode contacts over auditory areas in the STG mainly responded to the voice, and those over the visual FG mainly to the face stimulus. Moreover, ATL responses to the voices or faces tended to be in lower frequency bands (strongest in the **beta band**), whereas unisensory responses in the STG and FG were in the **gamma band** (Figure 4). This might be of interest in relation to suggestions that gamma is a measure of local or feed-forward processes, while beta band activity could be an indication of top-down feedback [78]. One speculative possibility is that the ATL is receiving and providing face and voice feedback on unisensory cortex, consistent with cognitive models whereby the ATL reactivates [15] or routes information held in sensory-specific cortex. Alternatively, even certain feed-forward processes in the ATL might not appear in the gamma range because the temporal coordination of neural activity generating oscillations may differ across regions. Tentatively, these human intracranial recording results suggest modality-independent representations in parts of the ATL, while sensory-specific responses dominate in the superior (voice) and inferior (face) portions of the ATL. However, given that the task in these human studies involved lexical retrieval, it remains important to assess face- and voice-sensitive processes using nonlinguistic tasks.

## Establishing Better Causal Relations with Identity Perception

Thus far, our understanding of how affecting neuronal processes or brain regions impacts on identity-related perception is limited. For practical reasons, recordings in monkeys and many human imaging studies are conducted with passive stimulation or a stimulus-irrelevant task, such as visual fixation. An earlier study showed that microstimulation of monkey IT neurons influenced subsequent face category judgments [97]. Recently, human transcranial magnetic stimulation of temporal voice regions selectively disrupted voice category judgments [98]. In another study, directly stimulating the FG of a human patient warped the patient's perception of a face [99]. Whether these manipulations would have also affected perception in another sensory modality from the one studied is a topic for future research.

## A Primate Model of Identity Processes

**Figure 5:**
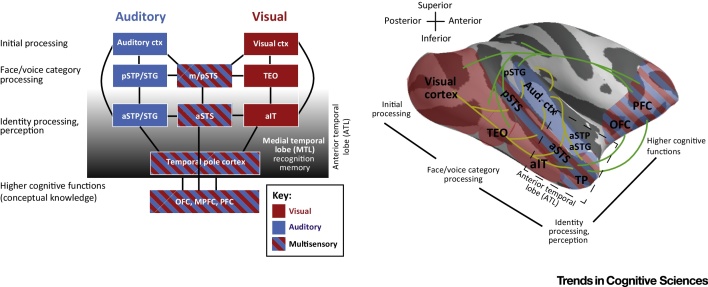
Key Figure: Primate Model for Identity-Processing and Multisensory Convergence

We propose a model of individual identity processes in primates, on the basis of the prior synthesis (Figure 5, Key Figure), as follows: (i) two independent but interacting auditory and visual ventral processing streams extract voice or face features. ATL regions are sensitive to identity features, with other temporal lobe regions evaluating different aspects of voice or face content, such as category membership; (ii) the STS is a key conduit between voice and face processing streams, with the aSTS an ATL convergence site that allows multisensory representations to more strongly influence frontal cortex; (iii) neurons in ATL subregions, such as the aSTS and the temporal pole, integrate highly subcategorized information specific for unique individuals and concrete entities. Such representations may not be tied to any sensory modality and the neural mechanisms need to be determined (Box 1). Possibly, the ATL can feed back to unisensory processing streams to route specific forms of input; (iv) anatomical connectivity between the primate ATL regions is funneled into the temporopolar cortex 85, 100, but less is known about its functional role in primates in relation to identity processes; and (v) identity recognition is likely to involve MTL structures. Currently, it is an open question whether auditory pathways to the MTL in primates are less direct than those in humans 101, 102, requiring cross-species comparisons of interconnectivity.

The primate model at a regional level is generally in agreement with human models on face and voice perception, whereby distinct sensory processing streams have prominent multisensory interactions between face and voice areas 4, 5, 12. One issue that needs addressing is whether human voice regions in the STG and STS are intrinsically more multisensory than the voice regions in the primate aSTP. It is possible that human auditory voice regions in the STG are difficult to distinguish from neighboring multisensory regions in the STS in group neuroimaging data. Thus, the anterior upper bank of the STS may be a key site of multisensory convergence in both humans and monkeys. The model suggests that candidate regions for convergence sites in the ATL are the aSTS and the temporopolar cortex.

Furthermore, the multisensory computations underlying identity identification remain unclear. First, it is possible that, in certain ATL sites, a process resembling convergence on a larger scale might, at a finer scale, be found to be partially segregated by unisensory input 74, 77. Second, implicating either multisensory ‘synergistic’ versus ‘or gate’ mechanisms (Box 1) in specific regions cannot be resolved by current findings: while the monkey recordings from the uSTS appear consistent with a synergistic process, as suggested by results of nonadditive multisensory interactions, they also reveal independent activation by auditory or visual stimulation (Figure 3A [64]). The human ATL recordings that show strikingly similar responses to voice and face stimuli before naming, which differ from responses in unisensory regions [26], suggest an ‘or gate’ operation. In humans, when ATL voice- and face-responsive sites are injured, voice and face naming are both impaired [16], possibly suggestive of a synergistic interaction [16]. Formally testing the alternative neuronal mechanisms will require inactivating one of the input streams during multisensory stimulation, as we illustrate in Box 1, and might require animal models for adequate specificity.

While the notion of sites with amodal functions may well be disproved in the future, it is a useful concept for generating testable predictions on neuronal processes and multisensory interactions. It is also worth keeping in mind that the ATL is one of several highly multisensory convergence sites in the brain that serve various purposes. For example, the angular gyrus in humans is part of a multiple-demand, cognitive control network [103] that appears to also be present in monkeys [104]. There may also be a gradation between modality-specific and amodal representations in the ATL 19, 86, which our simple model does not capture but which could be explored with computational simulations as well as additional data on neuronal processes in convergence sites and those that influence them. Finally, the picture becomes more complex with feedback interactions, but are important to consider because cognitive ‘reactivation’ of the ATL during retrieval [15] may convert a synergistic process to an ‘or gate’.

## Identity Processes from a Broader Evolutionary Perspective

The proposed primate model may be generalized for testing in other nonhuman animals. Rodents identify each other by odor [105], and odor identity is represented in the olfactory piriform cortex 106, 107 (which is interconnected with the entorhinal cortex [108], one of the regions present in the primate MTL; Figure 5). Pup odors and vocalization sounds can synergistically interact to influence maternal behavior in mice [109], and there appear to be multisensory interactions between the rodent olfactory and auditory processing systems 110, 111, 112. Moreover, auditory object-identity processes (i.e., the timbre of resonant sources [116]) are being studied in ferrets [113], as is the distinction between the neuronal representation in songbirds of own song versus the song of another [114]. A broader comparative approach will clarify evolutionary relations and enable researchers to harness the strengths of different animals as neurobiological models.

## Concluding Remarks

By reviewing recent voice- and face-related neurobiological work in nonhuman primates and humans, we suggest here several principles that may be eventually extended for modeling the basic neural processes involved in subordinate or identity perception. The proposed model highlights some possible neural mechanisms and the key areas of uncertainty between the primate and human models. We argue that the next step in understanding the neurobiology of identity perception will benefit from cross-species comparisons, direct access to local neuronal processes in different ATL subregions, and causal manipulation of sensory inputs into convergence sites. We also need information on effective connectivity and to better establish causal relations between neuronal processes and identity perception and cognition (see Outstanding Questions). All such work will need to involve more than just one sensory modality.

Outstanding Questions Is any sensory input at multisensory convergence sites sufficient for the identification of conspecifics, or are all incoming signals integrated and identification emerges out of their synergistic interaction?

Which subregions of the human ATL support identity processes, how are they structurally and functionally interconnected, and how does this compare to data in animal models?

How do ATL regions functionally interact with other brain regions, and is interaction with medial temporal lobe structures required for identity recognition and memory?

Can animal studies clarify whether certain ATL processes are crucial for perception and/or conceptual knowledge? Attentional cuing tasks can be used to assess the perceptual awareness of an animal towards attended voice or face features. Also, person-specific conceptual knowledge could be modeled in nonhuman animals to assess modality-independent representations of specific individuals, using voices and/or faces of familiar animals or those with known social categories (e.g., social rank, etc.) within adaptation, oddball, or other experimental paradigms.

What is the impact of degrading or inactivating a sensory input stream on the neuronal responses at face–voice convergence sites?

Which subregions of the ATL have amodal representations of identity, and do these require an ‘or gate’ process or does top-down selection serve as the gating mechanism?

Regardless of the nature of the multisensory mechanisms, what is the pattern of encoding at the neuronal subpopulation level in any of these regions (e.g., distributed, sparse, or other coding strategy) and how is this affected by removing sensory inputs?

How do oscillatory mechanisms support identity representations in the ATL, and are these processes specifically involved in person identification or do they form part of general computational principles that apply across sensory tasks and brain regions?

## Figures and Tables

**Figure I:**
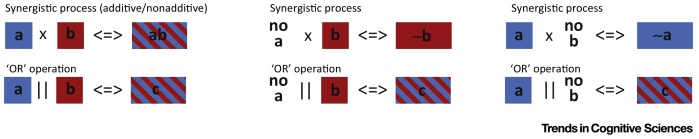
Illustration of How Different Multisensory Neural Mechanisms Could be Dissociated by Eliminating One Form of Sensory Input (a or b).

## Box 1 Predicted Neuronal Mechanisms and Impact of Lost Unisensory Input

Multisensory convergence sites have responses that are influenced by the unisensory streams feeding into the region, but the neuronal mechanisms for these convergence processes could be very different. Two simple mechanisms are illustrated in Figure I, as is the expected impact of lost unisensory function on neural responses at the convergence site. If the two inputs (‘a’ and ‘b’) are **additive** 120, 122, **multiplicative**, or **divisive neuronal responses**, the convergence site will reflect a synergistic combination of the two (‘ab’). Alternatively, if the convergence site functions as an ‘OR’ gate, then the result (‘c’) would differ from the form evident in the sensory inputs, as would a synergistic process (‘ab’), but the neuronal computations involved are different.

To tease apart the mechanism requires eliminating or degrading one form of input (such as by using local, reversible molecular or genetic neuronal inactivation in an animal model) while stimulating with multisensory input (e.g., voice and face of a specific individual). Then, assessing whether the convergence site shows more ‘a’ or ‘b’ responsiveness would clarify whether the mechanism is a synergy that is disrupted if one input stream is lost. The alternative is that the loss of input from one sensory stream does not qualitatively alter the form of the responsiveness in the convergence site. It is currently unknown which of these, or other, mechanisms are implemented in any of the multisensory sites identified in Figure 5 (main text).

Related predictions can be extended to measures of oscillatory activity rather than firing rates, with the main difference being the patterns of combined multisensory responses at convergence sites: dominant sensory input typically elicits broadband power increase and strong low-frequency phase alignment, while nondominant crossmodal inputs can reset the phase of ongoing low-frequency cortical oscillations without a strong increase in power. Such cross-sensory phase resetting can predict multisensory enhancement or suppression of spiking responses depending on its phase relation to the dominant sensory response 71, 121. In intact synergistic processes, the multisensory oscillatory response resulting from a combination of dominant and nondominant inputs could be a scaled approximation of the response to the dominant inputs. By contrast, in an ‘OR’ operation, different equally dominant inputs may be combined into a form of oscillatory response that is characteristic of that particular multisensory site. Again, to tease apart the mechanism requires eliminating or degrading one form of sensory input.

## Glossary

Additive/multiplicative/divisive neuronal responses: multisensory interactions are measured when the response to combined sensory modalities differs from any of the responses to the different modalities in isolation. Additive responses are modeled as the sum of the individual sensory responses. Multiplicative or divisive responses are nonadditive, nonlinear multisensory responses.
Amodal: a transmodal or modality-free representation of an environmental object where input from any one or multiple sensory stream(s) can contribute towards identifying the object. Our definition does not require or imply a symbolic or semantic transformation. This is a type of multisensory representation, but unlike multisensory influences between sensory streams, losing one set of unisensory inputs will not preclude identification of the object by any of the other modalities.
Anterior temporal lobe (ATL): structures in and around the temporal pole in both hemispheres of the primate brain. This includes the temporal pole, aSTP, aSTG, aSTS, anterior middle TG (aMTG; a gyrus present in humans but not monkeys) and the aIT, which includes the inferior temporal gyrus (ITG) and in humans, the aFG. Medial aspects of the ATL include anterior parts of the amygdala and entorhinal cortex. Functionally distinct ATL modules can be parcellated based on cytoarchitectonics [115] and the sensory profiles of the afferent input streams and efferent projections to frontal areas [100]. Most temporal pole subregions appear to be more strongly interconnected with specific other ATL subregions, while the polar area, TG [115], is connected to all other areas of the temporal pole [85].
Beta band oscillations: brain rhythms that fluctuate in the approximately 15–30Hz range.
Depth electrode recordings: intracerebral recordings from deep cortical, sulcal and sub-cortical structures below the surface of the brain.
Electrocorticography (ECoG): also known as intracranial electroencephalography (iEEG), typically refers to intracranial recordings from the surface of the brain, as performed in patients with epilepsy being monitored for invasive localization of their epileptogenic foci.
Gamma band oscillations: electroencephalography or intracranial recordings can measure rhythmic oscillations thought to reflect the coordinated spiking activity of large groups of neurons. Gamma band oscillations occur above 30 Hz.
Intracranial recordings: direct extracellular electrical recordings from within the gray matter or the surface of the brain.
Multisensory convergence: neurons or brain areas receiving input from multiple sensory pathways, such that their responses are affected by inputs in any of the converging sensory modalities. Multisensory convergence is thought to be the basis for integrating different sensory inputs into a unified, multisensory representation, but might differ mechanistically from an amodal representation, as we consider in this article.
Neuroimaging: brain-imaging approaches measuring hemodynamic responses with fMRI or functional near-infrared spectroscopy (fNIRS), glucose metabolism with positron emission tomography (PET) or electrical (electroencephalography, EEG) or magnetic activity (magnetoencephalography, MEG) from the surface of the head.
Phonagnosia: a variant of auditory agnosia where a lesion impairs the ability to perceive or recognize the voice of an individual, often with preserved speech comprehension.
Prosopagnosia: a deficit where a person's ability to perceive and recognize faces is impaired, although their ability to perceive and recognize other objects may be intact. This can result from damage to the face-processing network in the temporal lobe. Prosopagnosia can, but does not necessarily always, dissociate from phonagnosia.
Selectivity: measure of the size of the stimulus set evoking responses from a neuron or set of neurons, as an indication of the broadness of tuning. This value can range from weakly selective neurons that respond to none or most of the presented stimuli to neurons responding to a subset of the stimuli but not the others.
Sensitivity: measure of the stimulus category that drives a neuron or set of neurons. For instance, a voice-sensitive neuron might respond strongly to different voices, but less to nonvoice sounds and, thus, would carry information about a ‘voice’ category. A voice identity-sensitive neuron would respond selectively to a subset of the voice category stimuli. An extreme case of identity sensitivity is the traditional notion of an identity-selective ‘grandmother cell’ that responds exclusively to one particular individual in a one-or-nothing fashion.
Voice or face content: the sensory features of vocalizations or faces that provide indexical cues to the identity of the individual. For example, several acoustical factors (including the vocal filtering of the sound generated by the vocal source in the mammalian larynx) could be used to identify an individual by the voice characteristics of their vocalizations. More generally, voice features are related to the identity (timbre) of resonant sources 113, 116.

## Resources

^i^ https://pysurfer.github.io/
